# Wild Edible Fruits as a Source of Food and Medicine: A Study among Tribal Communities of Southern Khyber Pakhtunkhwa

**DOI:** 10.3390/plants13010039

**Published:** 2023-12-21

**Authors:** Sheikh Zain Ul Abidin, Raees Khan, Mushtaq Ahmad, Alain Cuerrier, Muhammad Zafar, Asad Ullah, Jabbar Khan, Asma Saeed, Wahidah H. Al-Qahtani, Mohsin Kazi

**Affiliations:** 1 Institute of Biological Sciences, Gomal University, Dera Ismail Khan 29050, Pakistan; zain@gu.edu.pk (S.Z.U.A.); sjabbarkhan@yahoo.com (J.K.); asmasaeed@gu.edu.pk (A.S.); 2 Department of Plant Sciences, Quaid-i-Azam University, Islamabad 45320, Pakistan; mushtaqflora@hotmail.com (M.A.); zafar@qau.edu.pk (M.Z.); 3 National Herbarium, National Agricultural Research Centre, Pakistan Agricultural Research, Council, Islamabad 45500, Pakistan; 4 Biological Sciences Department, University of Montreal, Montréal, QC H2V 0B3, Canada; alaincuerrier@ville.montreal.qc.ca; 5 Centre of Plant Biodiversity, University of Peshawar, Peshawar 25120, Pakistan; asadcpb@uop.edu.pk; 6 Department of Food Sciences & Nutrition, College of Food & Agriculture Sciences, King Saud University, Riyadh 11451, Saudi Arabia; wahidahhalqahtani@gmail.com; 7 Department of Pharmaceutics, College of Pharmacy, King Saud University, P.O. Box 2457, Riyadh 11451, Saudi Arabia; mkazi@ksu.edu.sa

**Keywords:** edible wild fruits, food security, southern Khyber Pakhtunkhwa, nutraceutical, quantitative ethnobotanical indices (QEI), relative frequency citation (RFC), use value (UV)

## Abstract

Botanical surveys in all parts of Pakistan are mainly focused on ethnomedicinal uses of plants, and very little attention has been paid to documenting edible wild fruit species (EWFs). Multiple methodologies and tools were used for data collection. In a recent survey 74 EWF species belonging to 29 families were documented, including their medicinal uses for the treatment of various diseases. The most cited (23%) preparation method was raw, fresh parts. The UV and RFC of EWF species ranged from 0.08 to 0.4 and from 0.02 to 0.18, respectively. In terms of specific disease treatments and their consensus, the ICF ranged from 0 to 0.38. Sexual, gastrointestinal, and respiratory disorders had the highest use reports, and 11 species of plants had the highest FL of 100%. On the basis of uses reported by the inhabitants of seven districts of Southern Khyber Pakhtunkhwa Province, the CSI ranged from the lowest 1.3 to the highest 41. It is concluded that the traditional uses of EWF species depend mainly on socio-economic factors rather than climatic conditions or the number of species. However, there is a gradual loss of traditional knowledge among the younger generations. The present survey is the first baseline study about the socio-economic dimension of local communities regarding the use of EWF species for food as well as medicine.

## 1. Introduction

Ethnobotany is a multidisciplinary approach [1] and can be applied to select medicinal plants for pharmacological studies [2], as a precursor to drug development [3]. Since the dawn of human civilization, food and defense against various disease-causing pathogens have been the primary concerns [4]. Medicinal plants represent a large portion of the floristic richness worldwide [5]. Previous ethnobotanical surveys have mainly focused on traditional medicines based solely on medicinal plants [6,7], but very little attention has been given to edible wild fruit species (EWFs) [8,9]. However, over the last few years, an increasing interest in EWF species has been noticed among different communities of the world [10], and there is an incentive to rediscover the importance of traditional medicine based on EWF species [11]. The EWFs are generally characterized by high medicinal and nutritional values [12,13,14,15], a higher fiber content [9], and being rich in antioxidants and flavonoids [9]. Apart from their nutritional perspectives, many of them have yielded important beneficial outcomes in preventing and healing several chronic diseases, such as age-related disorders, heart disorders, diabetes, and various kinds of cancers [16,17]. Previous studies indicate that more than 300 million people throughout the world depend, for their livelihood, on forests abundant with EWF species [18,19]. This dependence is very common in areas where there is a rich diversity of these species with easy access for local communities [20].

With rapid economic growth, many traditional uses of EWF species are decreasing. The migration of people to urbanized areas, the adoption of new lifestyles, and other factors led to the replacement of traditional medicine with allopathic medicine. Factors like environmental fluctuations, earthquakes, natural disasters and anthropogenic activities have also influenced traditional knowledge. There is a high need to revive and conserve this valuable indigenous knowledge, but only limited studies have been conducted on the traditional uses of EWF species in different parts of the world, for example, in Italy [21], some other European countries [22], and Pakistan [23]. 

In Pakistan, there are about 6000 species of higher plants [24,25]. Of them, 400–600 are considered important because of their medicinal properties based on ethno-botanical-directed research [26]. However, little attention has been paid in the past to exploring their medicinal values as well as their nutritional perspectives. Abbasi et al. [23] stated that the traditional use of EWF species is declining. Similarly, only a few studies have been conducted on their phytochemical and biological properties in Pakistan [27]. Therefore, ethno-directed research can be useful in the documentation and identification of these species and aims at developing new drugs, generating food resources, and conserving the resources themselves [2]. 

The EWFs distributed in rural areas are vital sources of food and a valuable source of nutrition for the local communities. The current study aims (i) to enlist EWF species distributed in the tribal communities of Southern Khyber Pakhtunkhwa, Pakistan; (ii) to document local knowledge related to these EWF species; and (iii) to compare their uses with the available literature.

## 2. Materials and Methods

### 2.1. Study Area

**Figure 1:**
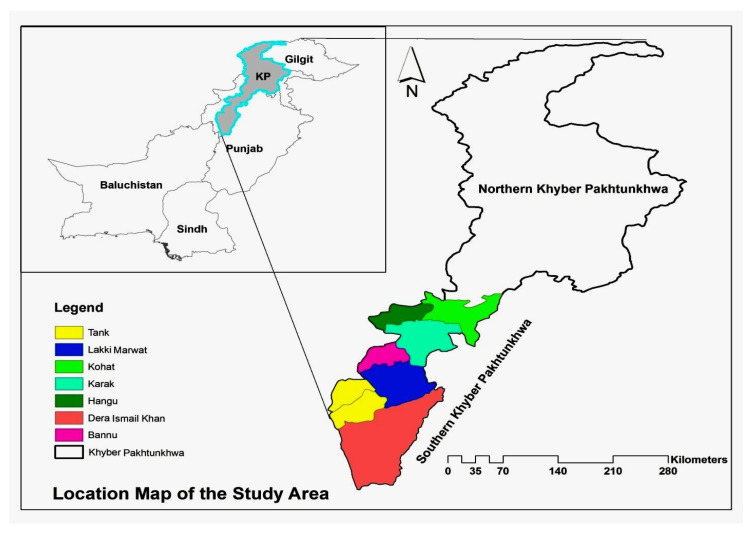
Map of study area.

The Khyber Pakhtunkhwa (KP) province (Pakistan) is located between 31°15″ and 32°32″ N and 70°11″ and 71°20″ E. It is divided into northern and southern parts. Southern KP is composed of Kohat, Karak, Hangu, Bannu, Lakki Marwat, Dera Ismail Khan, and Tank districts (Figure 1). In the west, it is bordered by the Orakzai and Kurram Agencies of the Federally Administrated Tribal Area (FATA) and it is bordered also by North and South Waziristan. In the east, it is linked to different districts of Southern Punjab. The Southern KP, where the project took place, is arid with hot summers and relatively mild winters, and scarcer in fall. Its climate varies from extremely cold to very hot. The maximum temperature in June has been recorded at 42 °C and the minimum at 27 °C in January [28]. The average rainfall varies from 70–90 mm in southern parts and 100–130 mm in northern parts. The main tribes of the Southern KP are Saraiky, Marwat, Yusufzai, Khattak, Shinwari, Bangash, Mahsud, Wazir, Syeds, Awans, Qureshis, Sardars, and Sheikhs.

Economically, Southern KP is lagging behind compared with Northern KP. The socio-economic features of informants show that the lower socio-economic class have no permanent source of income. Topography and a shortage of water are the main restraints for agriculture. Arable areas are restricted to 18.8% of the total land area. Rain-fed agriculture is mostly practiced in this region, and only 11% of the total cultivated area is irrigated [29].

### 2.2. Ethno-Medicinal Data Collection

The present study was conducted in 21 localities in South West Pakistan, for which the ISE code of ethics was followed by the International Society of Ethnobiology, [30]. In total, 233 local informants were interviewed using semi-structured questionnaire methods as employed by [31,32,33] and the guidelines endorsed by the International Society of Ethnobiology [30]. A total of 8 field surveys, each of which consisted of 7 to 8 days were established in the study area. In each field survey, participants were chosen randomly, except for key informants and Traditional Health Practitioners (THPs). Key informants are those who are experts about EWF localities and have experience using them. THPs were selected based on their experience and expertise.

During interviews, information about the local name of the plant, part(s) used, medicinal uses, disease treatment, and methods of preparation were documented by showing fresh specimens or photographs during field walks whenever possible to informants. Data about the demographic information of participants were also collected: age, gender, educational status, and experience with EWF uses. In most cases, data were cross-checked among informants of different villages, either by showing the fresh specimen, telling them about local names, or showing field photographs of wild fruit plants to verify the authenticity of the claims and select potential medicinal wild fruit plants of the study area.

### 2.3. Plant Identification and Comparative Studies

During fieldwork, 74 medicinally important EWF species were collected, pressed, and dried for correct taxonomic identification in the herbarium. All species were identified using the Flora of Pakistan [34,35]. After complete identification, plant specimens were submitted to the Herbarium of Pakistan (ISL), Quaid-i-Azam University Islamabad, for future reference. Further, botanical names of species were verified using the International Plant Name Index [36]. In addition to this, we compared the documented uses of EWFs with the previously published literature about their medicinal uses. The objective of this comparison was to underline changes that may have occurred between the literature and current medicinal data and also to assess the most important species for future phytochemical, pharmaceutical, and nutritional screening.

### 2.4. Quantitative Analysis of Ethnomedicinal Data

#### 2.4.1. Informant Consensus Factor (ICF)

The Informants’ Consensus Factor (ICF) [37,38] is determined using the following formula:

$$ICF=N_{ur}-N_{t}/(N_{ur}-1)$$

where “Nur” is the total number of use reports for each disease category and “Nt” represents the number of species used in that category. The ICF gives information about the consensus of informants about the use of EWFs and determines the consensus in the selection of EWFs against reported diseases. The maximum value that is close to 1 indicates that relatively well-known species are used by a large proportion of local people due to their authenticity in disease management, while a low value that is close to 0 shows that the informants disagree on the specificity of species and use any species by random selection to treat reported diseases [39].

#### 2.4.2. Use Value (UV) 

This quantitative method demonstrates the relative importance of plant species based on traditional uses. It is calculated according to the following formula:

UV=ΣU/N

where “UV” represents the use value of individual species; “U” represents the number of uses recorded for a species; and “N” represents the number of informants who reported species [38,40].

#### 2.4.3. Relative Frequency of Citation (RFC)

RFC shows the local importance of each species in the study area based on the number of informants [41,42]. This index is calculated using a formula from [43]. It varies between 0 and 1. It is calculated as follows:

RFC=FC/N(0<RFC<1)

where RFC stands for the relative frequency of citations; FC (frequency of citations) expresses the number of total informants for each species; and “N” shows the total informants interviewed in the study.

#### 2.4.4. Cultural Significance Index (CSI)

This is a quantitative anthropological technique in ethnobotany introduced by Turner [44] and recently changed by Da Silva et al. [45]. It describes the importance of plant species by assigning multiple ranking factors: species management (i), preference of species for a given use (e), and use frequency (c). A consensus called Correction Factor (CF) is also used to reduce the sensitivity of this method to sampling intensity. A value is given to each factor and ranks between 2 and 1. It is calculated using the following formula:

$$CSI=\sum_{i=1}^{n}(i=1=1)\times CF$$

CSI equates the Cultural Significance Index, “i” represents species management, “e” represents the preference of species to a given use, and “c” expresses the use frequency of each species, while CF stands for the correction factor. It is calculated by dividing the number of informants interviewed (FC) for each species by the maximum number of informants for any species. 

#### 2.4.5. Fidelity Level

Fidelity level (FL) expresses the preference for a species over others to treat any specific disease [46]. It is meant to select the most ideal species used in the treatment of a specific ailment [47]. It is calculated according to the following formula:

$$FL=I_{p}/I_{u}\times 100\;$$

where “Ip” represents the number of informants who mentioned the use of a species for a specific ailment, while “Iu” indicates informants who mentioned the species for any disease. 

## 3. Results

### 3.1. Demographic Data of Participants

The demographic information of the 233 participants interviewed (187 men and 46 women) is presented in Table 1. Male participants can be further divided into 163 indigenous lay-people and 24 Traditional Health Practitioners (THPs). In this study, male participants were greater in number than female participants due simply to the fact that women were reluctant to converse with male strangers (the interviewers). During the conversation with THPs, it was noted that they were highly interested in using EWFs for the treatment of various diseases as well as for nutritional purposes. The informants were divided into four different age groups, ranging from 18 to 80 years. The indigenous knowledge regarding the use of EWFs for the treatment of various ailments was more prevalent among the old participants, while the young ones were less knowledgeable. The reason might be the rapidly changing lifestyle and migration of the rural population to urbanized areas. Informants with knowledge about EWFs had a variety of different backgrounds and included retired army personnel, farmers, herdsmen, craftsmen, shopkeepers, teachers, and housewives. Most of the informants interviewed were educated up to secondary school education level or even less (Table 1). The local language is Saraiki or Pashto. Highly educated people were found to have less knowledge about the medicinal uses of EWFs as compared to illiterate and less educated people.

### 3.2. Taxonomic Diversity of EWFs

**Figure 2:**
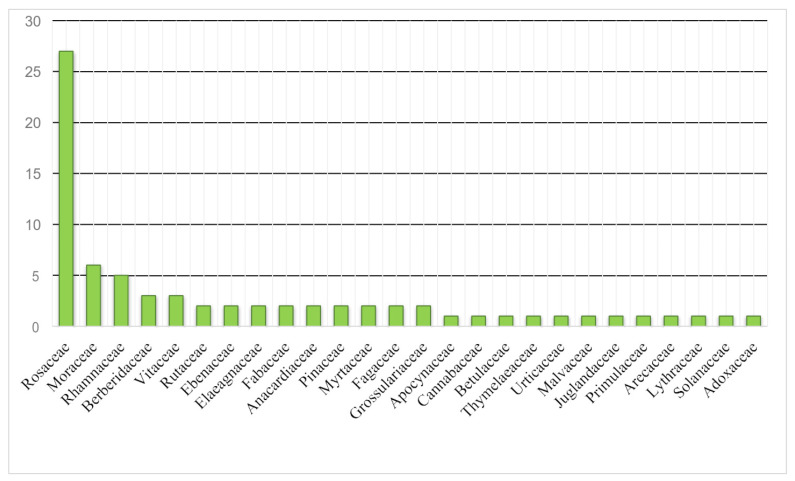
Families of wild edible fruits used in communities of Southern Khyber Pakhtunkhwa Province (Pakistan).

**Figure 3:**
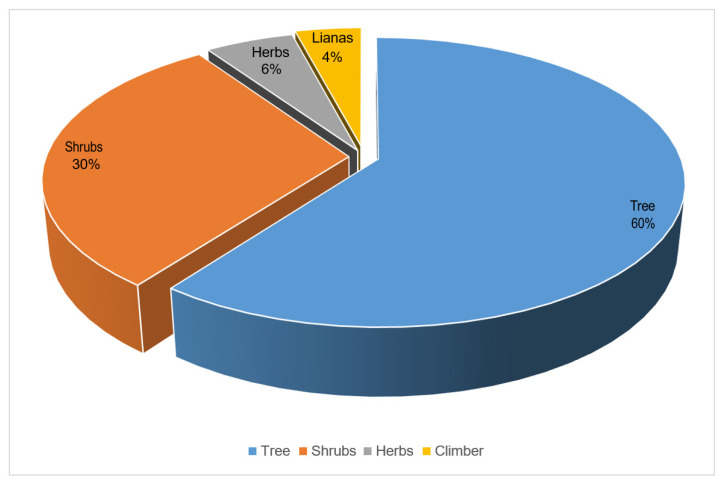
Life forms of wild edible fruits used in communities of Southern Khyber Pakhtunkhwa Province (Pakistan).

**Figure 4:**
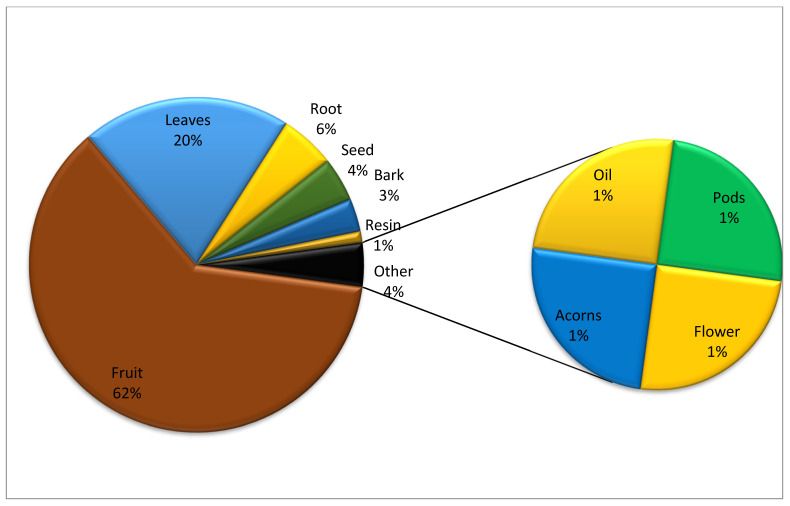
Plant parts used in treatment of various diseases.

**Figure 5:**
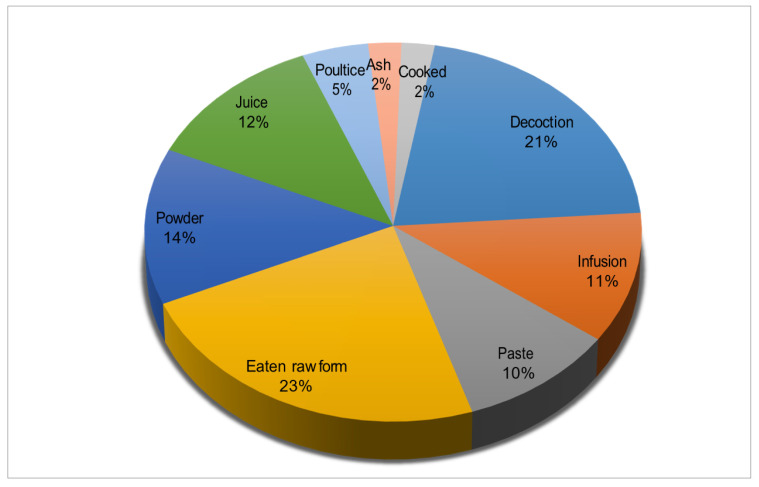
Mode of utilization of wild edible fruits of study area.

In this study, 74 EWF species belonging to 26 families were documented and assessed using ethnobotanical and quantitative techniques. Rosaceae was found to be the most cited family (27 species), followed by Moraceae (6 species) and Rhamnaceae (5 species) (Table 2 and Figure 2). Moraceae and Rhamnaceae were also rich in edible plant species (Figure 3). A total of 60% of the reported EWFs were trees, followed by shrubs (30%), and most fruits were obtained from trees. The present study shows (Figure 4) that the fruits were more frequently used. The present study reported nine preparation methods (Figure 5). These indigenous formulations were mostly prepared from single species, and mixtures were rare. Water was mostly used as a medium for preparation. On the other hand, milk, ghee, oil, eggs, and butter were used for applications in the majority of cases. The most cited preparation method was raw, fresh parts (23%), followed by decoctions (21%). The frequent use of fresh parts may be an artifact due to the aim of this study, i.e., documenting the use of wild fruits. The majority of EWFs in fresh form were sweet and delicious in taste, and they were eaten in raw form for treating different diseases. Eating whole, wild edible fruits was preferred because they are rich in energy and metabolites that play a great role in indigenous treatments. Decoctions were the second-most common preparation.

### 3.3. Informant Consensus Factor (ICF)

**Figure 6:**
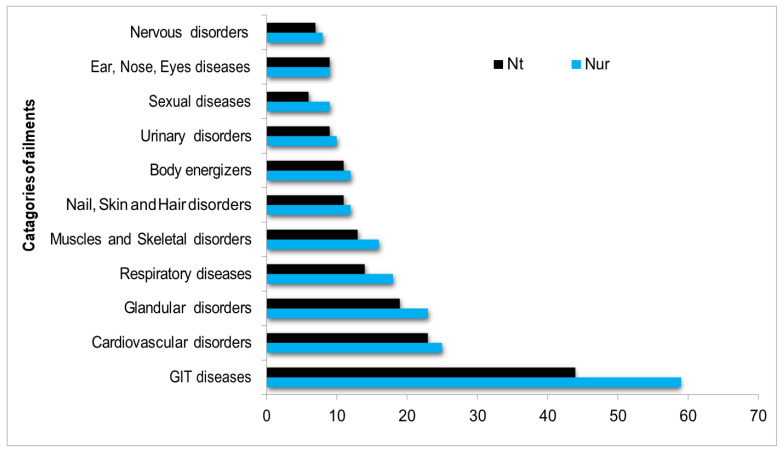
Categories of ailments treated by health practitioners arranged by number of use reports.

The ICF calculated for disease categories indicates the extent of homogeneity of the consensus among local people regarding the use of EWFs. In this study, the reported ailments are separated into 11 categories for ICF calculation and interpretation. The results show that the ICF values range from 0 to 0.38 (Table 3). The highest ICF value was observed for sexual disorders (0.38), followed by GIT diseases and respiratory diseases with 0.26 and 0.24, respectively. The use categories with more than 20 use reports were GIT diseases (59 use reports, 44 species), cardiovascular disorders (25 use reports, 23 species), and glandular disorders (23 use reports, 19 species) (Figure 6). The highest values of ICF in sexual, GIT, and respiratory disorders indicate that the inhabitants of select specific EWFs to treat those ailments. The top-ranked EWF species based on FC, reported for sexual disorders, were *Phoenix dactylifera*, *Celtis australis*, *Daphne mucronata*, and *Punica granatum*. In previous pharmacological studies, *D. mucronata* was not found as a remedy against sexual disorders.

*Phoenix dactylifera* and *Punica granatum* are common herbal sources that may be used against these disorders. The well-known species for GIT disorders were as follows: *Berberis aristata*, *Cydonia oblonga*, *Lathyrus aphaca*, and *Morus nigra*. *Cydonia oblonga* was reported in the current study. *Lathyrus aphaca* is reported to be effective against diarrhea and dysentery, but it has not yet been studied in pharmacological assays. For respiratory disorders, the most cited EWFs in this study were as follows: *Cydonia oblonga*, *Diospyros kaki*, *Ficus palmata*, *Prunus persica*, *Pyrus sinensis,* and *Solanum surattense*.

### 3.4. Fidelity Level (FL)

**Figure 7:**
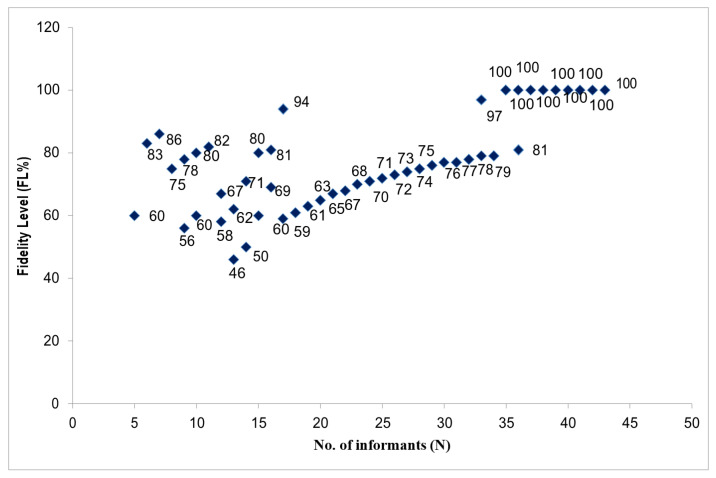
The relationship between the number of informants that mentioned use of a certain wild fruit for a particular disease; N is the total number of informants that cited the species for any disease. Numbers represent the fidelity level (FL %) as they appear in Table 3.

The relative healing potential of EWFs used against human ailments can be estimated using a fidelity level (Table 2). It determines which EWFs are used more, preferably for any specific ailment. The FL percentage varies from 46% to 100%, the highest value (Figure 7). Three categories of EWFs based on their FL percentage that inform us about their healing potential and popularity can be distinguished: well-known EWFs, moderately known EWFs, and little-known EWFs, with a range of FL percentages from 100 to 81, 80 to 61, and 60 to 46, respectively. The first category included 20 species, the second included 46 species, and the third included only 8 species (Figure 7). The well-known EWFs had 100% FL, underlining the choice made by local users to treat an ailment with a specific EWF. This pinpoints the curative properties of EWFs. A few species found in this study with 100% FL received attention in clinical trials and pharmacological assays. The present study revealed that *B. aristata* had the potential to treat constipation, and it should be further studied in detail. The current results also showed that *Daphne mucronata*, *Eriobotrya japonica*, *Ficus racemosa*, *Lathyrus aphaca*, *Malus pumila*, and *Morus nigra* were highly reported for the treatment of muscle pains, hypertension, diabetes, nerve problems, diabetes, and constipation.

### 3.5. Use Value (UV) and Relative Frequency of Citation (RFC)

**Figure 8:**
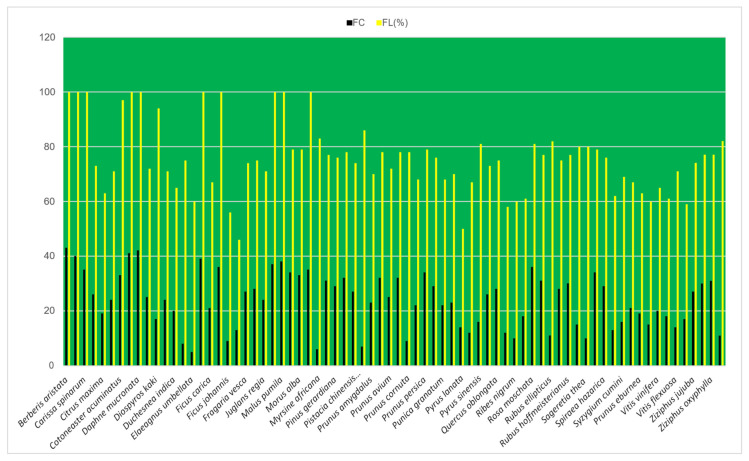
FC and FL (%) of wild edible fruit species.

**Figure 9:**
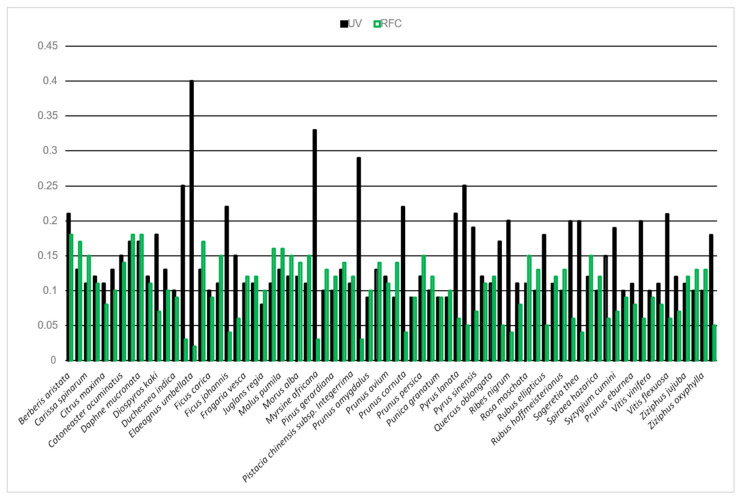
Quantitative analysis of wild edible medicinal fruits showing RFC and UV.

This index explains the prominence of the EWF species on the basis of their uses and the informants who reported these species (Figure 8). The UV ranges from the lowest 0.08 (*Juglans regia*) to the highest 0.4 (*Elaeagnus umbellata*). EWF species are classified into five classes based on the UV obtained: UV 0.08 to 0.10 (18 species), 0.11 to 0.12 (24 species), 0.13 to 0.17 (13 species), 0.18 to 0.25 (16 species), and UV from 0.26 to 0.4 (3 species). The species with the highest UV indicate common occurrence and utilization by the local people. Unlike UV, the RFC shows the local importance of the EWF species based on the relative informant ratios. In this study, it varies from the lowest 0.02 (*Elaeagnus umbellata*) to the highest 0.18 (*Berberis aristata*, *Cydonia oblonga*, and *Daphne mucronata*). Based on their prominence, the EWFs of this study were grouped into five RFC classes: 0.02 to 0.05 (12 species), 0.06 to 0.10 (25 species), 0.11 to 0.13 (18 species), 0.14 to 0.15 (12 species), and 0.16 to 0.18 (7 species). The species with the highest RFC were the most popular plants based on the majority of informants (Figure 9).

### 3.6. Cultural Significance Index

**Figure 10:**
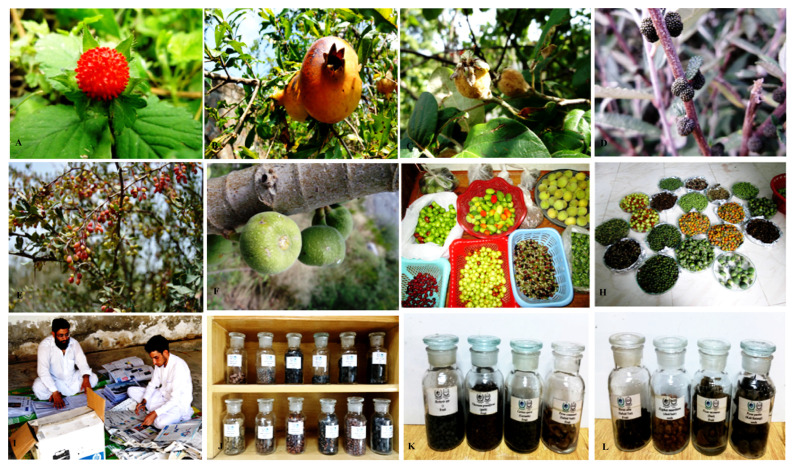
Edible wild fruits: *Fragaria nubicola* (**A**), *Punica granatum* (**B**), *Cydonia oblonga* (**C**), *Debregeasia salicifolia* (**D**), *Berberis lycium* (**E**), *Ficus racemosa* (**F**), collection (**G**,**H**), processing and identification (**I**), and its herbarium deposition (**J**–**L**).

To assess the importance of EWF species in local culture, the Cultural Significance Index (CSI) is calculated. On the basis of uses reported by inhabitants of seven districts of Southern KP, the CSI ranges from the lowest 1.3 to the highest 41. The top EWF species along with their specific uses were as follows: (jaundice), *Berberis lycium* (eye infections), *Cotoneaster acuminatus* (lung disorder), *Cydonia oblonga* (liver disorders), *Daphne mucronata* (muscle pain), *Eriobotrya japonica* (high blood pressure), *Malus pumila* (diabetes), *Prunus domestica* L. (constipation), *Ficus racemosa* (diabetes), *Morus nigra* (constipation), and *Rosa moschata* (skin diseases) (Figure 10).

### 3.7. Comparison with Previous Ethnomedicinal Studies

To compare to other studies, we used 21 available references (Table 2). Overall, 88.6% of the uses found in the current study represent new ethnobotanical data, while 5.6% of the uses and 5.8% of the reports were comparable to the literature. The EWFs found already in the literature were as follows: *Punica granatum*, *Cydonia oblonga*, *Berberis aristata*, *Berberis lyceum*, *Juglans regia*, *Ficus racemosa*, *Zanthoxylum armatum*, *Syzygium cumini*, and *Ziziphus jujuba*. This comparison shows that the majority of EWFs reported in the current study are confined to the topographical region of K. Some EWFs have, however, a wide distribution and are to be found in adjacent countries with similar medicinal uses. Nonetheless, among those species, some new medicinal uses were observed.

### 3.8. Threats to EWFs in Southern

The EWFs used by the tribal communities of Southern KP, Pakistan, are facing threats in their natural habitats, mainly due to anthropogenic activities. Such impacts vary, however, from place to place. According to some experienced THPs and key informants, overgrazing, overharvesting, uncontrolled fire setting, agricultural land expansion, roads and home construction, fodders, and fuel wood collection are some common threats to EWF species in the area. Most of the EWFs in the study areas are not protected. However, economically important species are overharvested and sold in the local herbal markets at very cheap prices. Some species are then traded to pharmaceutical companies.

## 4. Discussion

The results of the present study diverged from previously reported ethnobotanical studies conducted in Pakistan [24,25]. In most previous ethnobotanical studies, herbs were found to be the most commonly used [67,68,69,70,71,72,73]. Fruits were more frequently used instead of leaves, as observed in previous studies conducted in Pakistan [24,25,26] as well as all over the world [74,75,76,77,78]. The majority of EWFs were reported as sweet and delicious in taste [22]. Decoctions, the second-most common preparation, were reported in the literature as the most used preparation method in previous ethnomedicinal studies in Pakistan and other regions of the world [47,79,80].

Medicinal plants that are presumed to be more effective for treating certain diseases should have higher ICF values [81,82]. In this study, *Cydonia oblonga* was used for gastro-intestinal disorders. *Cydonia oblonga* was also reported by Romero et al. [83] against diarrhea, dysentery, and gastric ulcers. *B. aristata* was studied by Joshi et al. [84] to treat stomach infections and ulcers with inconclusive results, but the potential of this plant for treating piles and constipation has never been studied. Also, Gilani and Janbaz [85] assessed the hepatoprotective effect of *B. aristata*. In a previous study, Tosun et al. [86] evaluated the antimicrobial activity of *C. oblonga* to treat tuberculosis, whereas *D. kaki*, *F. palmata*, and *P. sinensis* have no previous pharmacological record against respiratory disorders.

A few species found in this study with 100% FL received attention in clinical trials and pharmacological assays. Joshi et al. [84] studied the antidiarrheal activity of *Berberis aristata.* The same species was evaluated for hepatotoxicity by [85]. The present study revealed that *B. aristata* had the potential to treat constipation, and it should be further studied in detail. Another top-ranked plant, *Carissa spinarum*, has been studied against hepatitis [87], internal infections [88], and heart problems [89], but no study has been performed in relation to asthma, a key finding in this study. *D. mucronata*, *L. aphaca,* and *M. nigra* have not been scientifically studied in detail, while the other species have received attention in pharmacological studies [90,91]. A very high FL may give indications about which species to prioritize in pharmacological, phytochemical, and clinical studies for the reported specific uses cited in the current survey [92]. The UV and RFC can be used to select potential candidates among all plant species for further pharmacological studies and, therefore, state recommendations for drug discovery and development. Species that have been studied using pharmacological assays are for stomach infections and ulcers [84], eye infections [93], jaundice [85], and wounds and skin diseases [94].

To compare to other studies, 21 available reference studies were used (Table 2). Overall, 88.6% of the uses found in this study represent new ethnobotanical data, while 5.6% of the uses and 5.8% of the reports were comparable to the literature. The EWFs found already in the literature were as follows: *Punica granatum*, *Cydonia oblonga*, *Berberis aristata*, *Berberis lyceum*, *Juglans regia*, *Ficus racemosa*, *Zanthoxylum armatum*, *Syzygium cumini*, and *Ziziphus jujuba*. This comparison shows that the majority of EWFs reported in the current study are confined to the topographical region of KP. Some EWFs found have, however, a wide distribution and are to be found in adjacent countries with similar medicinal uses. The possible reason behind the dominance of Rosaceae may be due to the rich diversity of EWF species within the family, a diversity known and used by local inhabitants. Nonetheless, among those species, some new medicinal uses were observed. The use of *Ziziphus jujuba* for the treatment of diabetes is new to the literature, as is its use as a body tonic [64]. Economically important species are overharvested and sold in the local herbal markets at very cheap prices. Some species are then traded to pharmaceutical companies. The conservation of locally important and threatened species must be promoted in the future [95,96].

Although the current study enlists EWFs and their medicinal uses with little attention to documenting the ecological aspects or conservation status of the species. Further investigation of the nutritional value of these species is needed. The current exploration deals with data collection from specific localities, and a more comprehensive study is required to enlist all EWFs and their traditional uses in Pakistan. 

## 5. Conclusions

The ethnobotanical data obtained from this survey show that the inhabitants of KP have a close relationship with EWF species. The tradition of using EWFs is very much alive among the different populations of Southern KP, and a total of 74 EWF species are extensively consumed as food as well as medicine. The number of recorded EWFs and their medicinal uses indicate the depth of the local indigenous knowledge of medicinal plants and their application. This provides evidence that EWFs continue to play a vital role in the health care systems of the indigenous people in the study area. Our study also underlines that the pattern of EWF usage depends mainly on socio-economic factors rather than climatic conditions or its floristic diversity.

## Figures and Tables

**Table 1:** Demographic data of participants.

Variable	Categories	No. of Persons	Percentage
Informant category	Traditional health practitioners	24	10.3
	Indigenous people	209	89.7
Gender	Female	46	19.7
	Male	187	80.3
Age	Less than 20 years	25	10.7
	20–30 years	34	14.6
	30–40 years	38	16.3
	40–50 years	40	17.2
	50–60 years	44	18.9
	More than 60 years	52	22.3
Educational background	Illiterate	47	20.2
	Completed five years education	38	16.3
	Completed eight years education	44	18.9
	Completed 10 years education	36	15.5
	Completed 12 years education	29	12.4
	Some under r grade degree (16 years education)	25	10.7
	Graduate (higher education)	14	6.0
Experience of the traditional health practitioners	Less than 2 years	6	25.0
	2–5 years	9	37.5
	5–10 years	4	16.7
	10–20 years	3	12.5
	More than 20 years	2	8.3

**Table 2:** Medicinal uses of EWFs in Southern KPK province, Pakistan.

Family Name	Botanical Name	Voucher Number	Local Name	Life Form	Part(s) Used	Mode of Utilization	Medicinal Uses	FC	RFC	UR	UV	FL (%)	CSI	Recorded Literature Used
Adoxaceae	*Viburnum grandiflorum* Wall. ex DC.	ISI-HS-40	Guch	Tree	Fruit, Seed	Juice, Poultice	Typhoid, cough, fever	15	0.06	3	0.2	60	4.6	[48]∆,[23]∆,[49]∆,[50]∆,[51]∆,[52]∆,[53]∆,[43]^®^,[54]∆,[55]∆,[56,57]∆,[58]∆,[59]^®^,[60]∆,[61]∆,[62]∆,[63]∆,[64]^®^,[65]∆,[66]∆
Anacardiaceae	*Mangifera indica* L.	ISI-HS-9	Aam	Tree	Leaves	Infusion	Diarrhea, dysentery, urethritis, diabetes	34	0.15	4	0.12	79	13.4	[48]∆,[23]∆,[49]^®^,[50]∆,[51]∆,[52]∆,[53]∆,[43]∆,[54]∆,[55]∆,[56,57]∆,[58]∆,[59]∆,[60]∆,[61]∆,[62]∆,[63]∆,[64]∆,[65]∆,[66]♦
	*Pistacia chinensis* subsp. *integerrima* (J. L. Stewart ex Brandis) Rech. f.	ISI-HS-2	Kangar	Tree	Fruit	Ash, decoction	Cough, dysentery, jaundice	27	0.12	3	0.11	74	9.5	[48]∆,[23]^®^,[49]∆,[50]∆,[51]∆,[52]∆,[53]∆,[54]∆,[43]∆,[55]∆,[56,57]∆,[58]∆,[59]∆,[60]∆,[61]∆,[62]∆,[63]∆,[64]∆,[65]∆,[66]∆
Apocynaceae	*Carissa spinarum* L.	ISI-HS-12	Garanda	Shrub	Fruit	Eaten raw form	Heart tonic, **asthma**, hepatitis and internal infections	35	0.15	4	0.11	100	13.8	[48]∆,[23]∆,[49]∆,[50]∆,[51]∆,[52]∆,[53]∆,[43]∆,[54]∆,[55]∆,[56]∆,[57]∆,[58]∆,[59]∆,[60]∆,[61]∆,[62]∆,[63]∆,[64]∆,[65]∆,[66]∆
Arecaceae	*Phoenix dactylifera* L.	ISI-HS-19	Khajoor	Tree	Fruit	Eaten raw	Brain tonic, aphrodiasc, **blood pressure**	31	0.13	3	0.1	77	9.4	[48]∆,[23]^®^,[49]∆,[50]∆,[51]∆,[52]∆,[53]∆,[43]∆,[54]∆,[55]∆,[56]^®^,[57]∆,[58]∆,[59]∆,[60]∆,[61]∆,[62]^®^,[63]∆,[64]∆,[65]∆,[66]∆
Berberidaceae	*Berberis aristata* DC.	ISI-HS-4	Zareshk, Sumbal	Shrub	Leaves	Decoction, Paste	Stomach infection, piles, ulcers, fever, constipation, eyes infection, **jaundice**, wounds, skin diseases	43	0.18	9	0.21	100	41	[48]♦,[23]∆,[49]∆,[50]∆,[51]∆,[52]♦,[53]∆,[43]∆,[54]∆,[55]∆,[56]∆,[57]∆,[58]∆,[59]♦[60]∆,[61]∆,[62]∆,[63]∆,[64]♦[65]∆,[66]∆
	*Berberis lycium* Royle	ISI-HS-34	Sumbal	Shrub	Root	Powder	Diarrhea, piles, **eyes infection**, internal wounds, external wounds	40	0.17	5	0.13	100	19.5	[48]∆,[23]^®^,[49]∆,[50]♦,[51]∆,[52]♦,[53]∆,[43]♦,[54]∆,[55]∆,[56]∆,[57]∆,[58]^®^[59]∆,♦,[60]∆,[61]∆,[62]∆,[63]∆,[64]^®^,[65]∆,[66]∆
	*Sinopodophyllum hexandrum* (Royle) T.S.Ying	ISI-HS-22	Bankakri	Herb	Root	Infusion	Blood purifier, diarrhea	7	0.03	2	0.29	86	1.4	[48]∆,[23]∆,[49]∆,[50]∆,[51]∆,[52]∆,[53]∆,[43]∆,[54]∆,[55]∆,[56]∆,[57]∆,[58]∆,[59]∆,[60]∆,[61]∆,[62]∆,[63]∆,[64]∆,[65]^®^,[66]∆
Betulaceae	*Corylus colurna* L.	ISI-HS-24	Urni	Tree	Seed	Eaten raw form	Body tonic, **fever**, nerve tonic	24	0.1	3	0.13	71	7.8	[48]∆,[23]∆,[49]∆,[50]∆,[51]∆,[52]∆,[53]∆,[43]∆,[54]∆,[55]∆,[56]∆,[57]∆,[58]∆,[59]∆,[60]∆,[61]∆,[62]∆,[63]∆,[64]∆,[65]∆,[66]∆
Cannabaceae	*Celtis australis* L.	ISI-HS-41	Batkarar	Tree	Leaves, Fruit	Decoction	Diarrhea, dyspepsia, amenorrhea	26	0.11	3	0.12	73	8.4	[48]∆,[23]∆,[49]∆,[50]∆,[51]∆,[52]∆,[53]♦,[43]∆,[54]∆,[55]∆,[56]∆,[57]∆,[58]∆,[59]∆,[60]∆,[61]∆,[62]∆,[63]∆,[64]∆,[65]∆,[66]∆
Ebenaceae	*Diospyros kaki* L.f.	ISI-HS-43	Japanese fruit	Tree	Fruit	Eaten raw	Stomachic, constipation, lungs disorder	17	0.07	3	0.18	94	5.6	[48]∆,[23]∆,[49]∆,[50]∆,[51]^®^,[52]∆,[53]∆,[43]♦,[54]∆,[55]^®^,[56]∆,[57]∆,[58]∆,[59]∆,[60]∆,[61]∆,[62]∆,[63]∆,[64]∆,[65]∆,[66]∆
	*Diospyros lotus* L.	ISI-HS-62	Kala Amlok	Tree	Fruit	Juice	Piles, eye infection, diarrhea	24	0.1	3	0.13	71	8.4	[48]∆,[23]^®^,[49]∆,[50]∆,[51]∆,[52]∆,[53]∆,[43]♦,[54]∆,[55]∆,[56]∆,[57]∆,[58]∆,[59]∆,[60]∆,[61]∆,[62]∆,[63]∆,[64]∆,[65]∆,[66]∆
Elaeagnaceae	*Elaeagnus angustifolia* L.	ISI-HS-I	Kankoli	Tree	Fruit	Decoction	Headache, arthritis	8	0.03	2	0.25	75	2.1	[48]∆,[23]∆,[49]∆,[50]∆,[51]^®^,[52]∆,[53]∆,[43]∆,[54]∆,[55]∆,[56]∆,[57]^®^,[58]∆,[59]∆,[60]∆,[61]∆,[62]∆,[63]∆,[64]∆,[65]^®^,[66]∆
	*Elaeagnus umbellata* Thunb.	ISI-HS-26	Kanrkoli	Tree	Fruit	Eaten raw	Cough, cardiac diseases	5	0.02	2	0.4	60	1.3	[48]∆,[23]∆,[49]∆,[50]∆,[51]∆,[52]∆,[53]∆,[43]♦,[54]∆,[55]∆,[56]∆,[57]∆,[58]∆,[59]∆,[60]∆,[61]∆,[62]∆,[63]∆,[64]∆,[65]∆,[66]∆
Fabaceae	*Tamarindus indica* L.	ISI-HS-48	Imli	Tree	Fruit	Juice	Jaundice, blood purification	21	0.09	2	0.1	67	4.9	[48]∆,[23]∆,[49]^®^,[50]∆,[51]∆,[52]∆,[53]∆,[43]∆,[54]∆,[55]∆,[56]∆,[57]∆,[58]∆,[59]∆,[60]∆,[61]∆,[62]^®^,[63]∆,[64]∆,[65]∆,[66]^®^
	*Lathyrus aphaca* L.	ISI-HS-35	Jangli matter	Herb	Pods	Eaten raw	Nerve tonic, diarrhea, dysentery, diuretic	37	0.16	4	0.11	100	14.6	[48]∆,[23]∆,[49]∆,[50]∆,[51]∆,[52]∆,[53]∆,[43]^®^,[54]∆,[55]∆,[56]∆,[57]∆,[58]∆,[59]∆,[60]∆,[61]∆,[62]∆,[63]∆,[64]∆,[65]∆,[66]∆
Fagaceae	*Quercus robur* L.	ISI-HS-20	Banchar	Tree	Oil	Paste	Skin diseases, **urinary disease**, muscular pain	26	0.11	3	0.12	73	9	[48]∆,[23]∆,[49]∆,[50]∆,[51]∆,[52]∆,[53]∆,[43]^®^,[54]∆,[55]∆,[56]∆,[57]∆,[58]∆,[59]∆,[60]∆,[61]∆,[62]∆,[63]∆,[64]∆,[65]∆,[66]∆,
	*Quercus oblongata* D.Don	ISI-HS-39	Barungi	Tree	Acorns	Cooked	Diuretic, diarrhea, dysentery	28	0.12	3	0.11	75	9.8	[48]^®^,[23]∆,[49]∆,[50]∆,[51]∆,[52]∆,[53]∆,[43]∆,[54]∆,[55]∆,[56]∆,[57]∆,[58]∆,[59]∆,[60]∆,[61]∆,[62]∆,[63]∆,[64]∆,[65]∆,[66]∆
Grossulariaceae	*Ribes himalense* Royle ex Decne	ISI-HS-33	Kag- Dakh	Tree	Leaves	Powder, Paste	External wounds, **Jaundice**	12	0.05	2	0.17	58	2.8	[48]∆,[23]∆,[49]∆,[50]∆,[51]∆,[52]∆,[53]∆,[43]∆,[54]∆,[55]∆,[56]∆,[57]∆,[58]∆,[59]∆,[60]∆,[61]∆,[62]∆,[63]∆,[64]∆,[65]∆,[66]^®^
	*Ribes nigrum* L.	ISI-HS-55	JangliDakh	Shrub	Fruit	Eaten raw	Hypertension, joint pain	10	0.04	2	0.2	60	2.3	[48]∆,[23]∆,[49]∆,[50]∆,[51]∆,[52]∆,[53]∆,[43]∆,[54]∆,[55]∆,[56]∆,[57]∆,[58]∆,[59]∆,[60]^®^,[61]^®^,[62]∆,[63]∆,[64]∆,[65]∆,[66]∆
Juglandaceae	*Juglans regia* L.	ISI-HS-60	Akhoer	Tree	Leaves, Fruit	Eaten raw	Weak teeth, cleaning teeth, **brain tonic**	24	0.1	2	0.08	71	5.6	[48]^®^,[23]^®^,[49]∆,[50]∆,[51]^®^,[52]^®^,[53]^®^,[43]^®^,[54]∆,[55]∆,[56]∆,[57]^®^,[58]∆,[59]^®^,[60]^®^,[61]^®^,[62]^®^,[63]^®^,[64]^®^,[65]∆,[66]∆
Lythraceae	*Punica granatum* L.	ISI-HS-5	Daruna	Tree	Fruit peel	Powder	Diarrhea, amenorrhea	22	0.09	2	0.09	68	4.6	[48]∆,[23]^®^,[49]^®^,[50]∆,[51]^®^,[52]^®^,[53]^®^,[43]^®^,[54]^®^,[55]^®^,[56]∆,[57]∆,[58]∆,[59]∆,[60]∆,[61]^®^,[62]^®^,[63]∆,[64]^®^,[65]^®^,[66]^®^
Malvaceae	*Grewia optiva* J.R.Drumm. ex Burret	ISI-HS-30	Damman	Tree	Leaves, Fruit	Decoction	Stomach, liver disorders, galactogogue	28	0.12	3	0.11	75	8.5	[48]∆,[23]^®^,[49]∆,[50]^®^,[51]∆,[52]∆,[53]^®^,[43]∆,[54]∆,[55]∆,[56]∆,[57]∆,[58]∆,[59]∆,[60]∆,[61]∆,[62]∆,[63]∆,[64]∆,[65]∆,[66]∆
Moraceae	*Ficus carica* L.	ISI-HS-45	Anjeer	Tree	Fruit	Eaten raw form	Anemia, constipation	21	0.09	2	0.1	67	5.4	[48]∆,[23]^®^,[49]∆,[50]∆,[51]^®^,[52]∆,[53]∆,[43]∆,[54]∆,[55]∆,[56]♦,[57]∆,[58]∆,[59]∆,[60]^®^,[61]♦,[62]^®^,[63]∆,[64]∆,[65]∆,[66]∆
	*Ficus racemosa* L.	ISI-HS-17	Rhumbal	Tree	Fruit	Infusion	Diabetes, liver disorders diarrhea, stomachic	36	0.15	4	0.11	100	15.1	[48]∆,[23]^®^,[49]∆,[50]∆,[51]∆,[52]∆,[53]∆,[43]∆,[54]∆,[55]∆,[56]∆,[57]∆,[58]∆,[59]∆,[60]∆,[61]∆,[62]∆,[63]∆,[64]∆,[65]∆,[66]∆
	*Ficus johannis* Boiss.	ISI-HS-63	TrekaniPhag	Tree	Fruit, Latex	Paste	Blood clotting, removal of thorns from skin	9	0.04	2	0.22	56	2.1	[48]∆,[23]∆,[49]∆,[50]∆,[51]∆,[52]∆,[53]∆,[43]∆,[54]∆,[55]∆,[56]^®^,[57]∆,[58]∆,[59]∆,[60]∆,[61]∆,[62]∆,[63]∆,[64]∆,[65]∆,[66]∆
	*Ficus palmata* Forssk.	ISI-HS-44	Phag	Tree	Fruit	Eaten raw	Lung diseases, constipation	13	0.06	2	0.15	46	2.7	[48]♦,[23]^®^,[49]∆,[50]∆,[51]∆,[52]∆,[53]♦,[43]♦,[54]∆,[55]∆,[56]∆,[57]∆,[58]∆,[59]^®^,[60]∆,[61]∆,[62]∆,[63]∆,[64]♦,[65]∆,[66]∆
	*Morus alba* L.	ISI-HS-59	Safeed toot	Tree	Leaves	Decoction	Kidneys, fatigue, **anemia**,galactogogue for cattle	33	0.14	4	0.12	79	13.9	[48]∆,[23]^®^,[49]∆,[50]∆,[51]∆,[52]∆,[53]^®^,[43]^®^,[54]^®^,[55]^®^,[56]∆,[57]∆,[58]♦,[59]∆,[60]^®^,[61]∆,[62]∆,[63]∆,[64]∆,[65]∆,[66]∆
	*Morus nigra* L.	ISI-HS-10	Kala toot	Tree	Leaves	Infusion	Constipation, fatigue, anemia, diuretic	35	0.15	4	0.11	100	14.6	[48]∆,[23]^®^,[49]∆,[50]∆,[51]∆,[52]∆,[53]^®^,[43]∆,[54]∆,[55]∆,[56]∆,[57]^®^,[58]^®^,[59]∆,[60]^®^,[61]∆,[62]∆,[63]^®^,[64]∆,[65]∆,[66]∆
Myrtaceae	*Syzygium cumini* (L.) Skeels	ISI-HS-15	Jamun	Shrub	Bark, Fruit	Powder, Juice	Dysentery, **diabetes**, jaundice	16	0.07	3	0.19	69	4.8	[48]∆,[23]∆,[49]^®^,[50]∆,[51]∆,[52]∆,[53]∆,[43]∆,[54]∆,[55]∆,[56]∆,[57]∆,[58]∆,[59]∆,[60]∆,[61]∆,[62]^®^,[63]∆,[64]∆,[65]∆,[66]♦
	*Psidium guajava* L.	ISI-HS-46	Amrood	Tree	Bark	Decoction	Diarrhea, **dysentery**, fever	29	0.12	3	0.1	76	10.1	[48]∆,[23]∆,[49]∆,[50]∆,[51]∆,[52]∆,[53]∆,[43]∆,[54]^®^,[55]∆,[56]∆,[57]∆,[58]∆,[59]∆,[60]∆,[61]∆,[62]∆,[63]∆,[64]∆,[65]∆,[66]^®^
Pinaceae	*Pinus gerardiana* Wall. ex D.Don	ISI-HS-58	Naezy	Tree	Seed	Eaten raw	Joint pain, heart tonic, jaundice	29	0.12	3	0.1	76	9.4	[48]∆,[23]∆,[49]∆,[50]∆,[51]∆,[52]∆,[53]∆,[43]∆,[54]∆,[55]∆,[56]∆,[57]∆,[58]∆,[59]∆,[60]∆,[61]∆,[62]∆,[63]∆,[64]∆,[65]^®^,[66]∆
	*Pinus roxburghii* Sarg.	ISI-HS-64	Cheer	Tree	Resin	Poultice	Antiseptic, diuretic, diaphoretic, discharging pus from wounds	32	0.14	4	0.13	78	13.3	[48]^®^,[23]∆,[49]∆,[50]♦,[51]∆,[52]∆,[53]^®^,[43]∆,[54]∆,[55]∆,[56]∆,[57]∆,[58]∆,[59]∆,[60]∆,[61]∆,[62]∆,[63]∆,[64]∆,[65]∆,[66]∆
Primulaceae	*Myrsine africana* L	ISI-HS-27	Khukan	Shrub	Fruit	Eaten raw	Diarrhea, anthelmintic	6	0.03	2	0.33	83	1.4	[48]∆,[23]^®^,[49]∆,[50]∆,[51]∆,[52]∆,[53]∆,[43]∆,[54]∆,[55]∆,[56]∆,[57]∆,[58]∆,[59]∆,[60]∆,[61]∆,[62]∆,[63]∆,[64]∆,[65]∆,[66]∆
Rhamnaceae	*Ziziphus jujuba* Mill.	ISI-HS-6	Beer	Tree	Fruit, Bark	Decoction, Eaten raw	Stomachic, **diabetes**, body tonic	27	0.12	3	0.11	74	8.2	[48]∆,[23]^®^,[49]∆,[50]∆,[51]∆,[52]∆,[53]^®^,[43]∆,[54]∆,[55]∆,[56]∆,[57]∆,[58]∆,[59]∆,[60]∆,[61]∆,[62]∆,[63]∆,[64]∆,[65]∆,[66]^®^
	*Ziziphus nummularia* (Burm.f.) Wight & Arn.	ISI-HS-38	Beeri	Tree	Fruit	Eaten raw	Blood purifier, body tonic, constipation	30	0.13	3	0.1	77	9.1	[48]∆,[23]^®^,[49]∆,[50]∆,[51]∆,[52]∆,[53]∆,[43]∆,[54]∆,[55]∆,[56]∆,[57]∆,[58]^®^,[59]∆,[60]∆,[61]∆,[62]∆,[63]∆,[64]∆,[65]∆,[66]∆
	*Ziziphus oxyphylla* Edgew.	ISI-HS-16	Phitni	Shrub	Fruit, Root	Juice	High blood pressure, jaundice, **gases**	31	0.13	3	0.1	77	10.1	[48]∆,[23]^®^,[49]∆,[50]∆,[51]∆,[52]∆,[53]∆,[43]∆,[54]∆,[55]∆,[56]∆,[57]∆,[58]∆,[59]∆,[60]∆,[61]∆,[62]∆,[63]∆,[64]∆,[65]∆,[66]∆
	*Ziziphus rugosa* Lam.	ISI-HS-3	Singli	Tree	Leaves	Powder	Diabetes, constipation	11	0.05	2	0.18	82	2.6	[48]∆,[23]^®^,[49]∆,[50]∆,[51]∆,[52]∆,[53]∆,[43]∆,[54]∆,[55]∆,[56]∆,[57]∆,[58]∆,[59]∆,[60]∆,[61]∆,[62]∆,[63]∆,[64]∆,[65]∆,[66]∆
	*Sageretia thea* (Osbeck) M.C.Johnst.	ISI-HS-29	Gangsseri	Shrub	Root	Infusion or Decoction	**Jaundice**, abdominal spasms	10	0.04	2	0.2	80	2.1	[48]∆,[23]∆,[49]∆,[50]∆,[51]∆,[52]∆,[53]∆,[43]∆,[54]∆,[55]∆,[56]∆,[57]∆,[58]∆,[59]∆,[60]∆,[61]∆,[62]∆,[63]∆,[64]∆,[65]∆,[66]∆
Rosaceae	*Cotoneaster acuminatus* Wall. ex Lindl.	ISI-HS-42	Luni	Tree	Fruit	Juice	Cardiotonic, diuretic, blood clots, blood purification, **lungs disorder**	33	0.14	5	0.15	97	16.2	[48]∆,[23]∆,[49]∆,[50]∆,[51]∆,[52]∆,[53]∆,[43]∆,[54]∆,[55]∆,[56]∆,[57]∆,[58]∆,[59]∆,[60]∆,[61]∆,[62]∆,[63]^®^,[64]∆,[65]∆,[66]∆
	*Cydonia oblonga* Mill.	ISI-HS-31	Phei	Tree	Fruit	Decoction	Tuberculosis, diarrhea, dysentery, gastric ulcer, **liver**, eye diseases, heart tonic	41	0.18	7	0.17	100	29.5	[48]∆,[23]∆,[49]∆,[50]∆,[51]♦,[52]∆,[53]∆,[43]∆,[54]∆,[55]∆,[56]∆,[57]♦,[58]∆,[59]∆,[60]♦,[61]♦,[62]♦,[63]♦,[64]∆,[65]∆,[66]∆
	*Duchesnea indica* (Jacks.) Focke	ISI-HS-32	Budemeava	Herb	Leaves, Fruit	Paste	Increasing blood circulation, **Skin diseases**	20	0.09	2	0.1	65	5.2	[48]^®^,[23]^®^,[49]∆,[50]∆,[51]∆,[52]∆,[53]∆,[43]∆,[54]^®^,[55]♦,[56]∆,[57]∆,[58]^®^,[59]∆,[60]∆,[61]∆,[62]∆,[63]∆,[64]∆,[65]∆,[66]∆
	*Eriobotrya japonica* (Thunb.) Lindl.	ISI-HS-8	Loquat	Tree	Leaves	Infusion	Heart tonic, hyperlipidemia, sedative, **hypertension**, diabetes	39	0.17	5	0.13	100	20	[48]∆,[23]∆,[49]∆,[50]∆,[51]∆,[52]∆,[53]∆,[43]∆,[54]∆,[55]^®^,[56]∆,[57]∆,[58]∆,[59]∆,[60]∆,[61]∆,[62]∆,[63]∆,[64]∆,[65]∆,[66]∆
	*Fragaria vesca* L.	ISI-HS-61	Budemeava	Shrub	Leaves Fruit	Decoction	Diuretic and refrigerant, **diarrhea**	27	0.12	3	0.11	74	8.2	[48]∆,[23]∆,[49]∆,[50]∆,[51]∆,[52]∆,[53]∆,[43]^®^,[54]∆,[55]∆,[56]∆,[57]∆,[58]∆,[59]∆,[60]^®^,[61]^®^,[62]∆,[63]∆,[64]∆,[65]∆,[66]∆
	*Malus pumila* Mill.	ISI-HS-25	Seb	Tree	Fruit	Eaten raw	Diabetes, cancer, constipation, dysentery, low blood pressure	38	0.16	5	0.13	100	19.4	[48]∆,[23]∆,[49]∆,[50]∆,[51]∆,[52]∆,[53]∆,[43]∆,[54]∆,[55]∆,[56]∆,[57]∆,[58]∆,[59]∆,[60]∆,[61]∆,[62]∆,[63]∆,[64]∆,[65]∆,[66]∆
	*Prunus amygdalus* Stokes	ISI-HS-65	Badam	Tree	Fruit	Powder	Rheumatism, nerve tonic	23	0.1	2	0.09	70	4.8	[48]∆,[23]∆,[49]∆,[50]∆,[51]∆,[52]∆,[53]∆,[43]∆,[54]∆,[55]∆,[56]∆,[57]∆,[58]∆,[59]∆,[60]^®^,[61]∆,[62]^®^,[63]∆,[64]∆,[65]∆,[66]∆
	*Prunus armeniaca* L	ISI-HS-11	Haare	Tree	Fruit	Decoction	Asthma, coughs, constipation, blood urination in cattle	32	0.14	4	0.13	78	13.3	[48]∆,[23]♦,[49]∆,[50]∆,[51]∆,[52]^®^,[53]∆,[43]^®^,[54]∆,[55]∆,[56]∆,[57]∆,[58]∆,[59]∆,[60]^®^,[61]∆,[62]∆,[63]∆,[64]^®^,[65]∆,[66]∆
	*Prunus avium* (L.) L.	ISI-HS-7	Kala kathi	Tree	Leaves, Fruit	Infusion	Diabetes, heart disease, eye Infection	25	0.11	3	0.12	72	7	[48]∆,[23]∆,[49]∆,[50]∆,[51]∆,[52]∆,[53]∆,[43]∆,[54]∆,[55]∆,[56]∆,[57]∆,[58]∆,[59]∆,[60]^®^,[61]^®^,[62]∆,[63]∆,[64]∆,[65]∆,[66]∆
	*Prunus domestica* L.	ISI-HS-70	Aloocha	Tree	Fruit	Infusion	Diarrhea, **constipation**, colic, nausea, yellow fever	32	0.14	3	0.09	78	15.5	[48]∆,[23]∆,[49]∆,[50]∆,[51]∆,[52]∆,[53]∆,[43]∆,[54]∆,[55]∆,[56]∆,[57]∆,[58]∆,[59]∆,[60]∆,[61]∆,[62]∆,[63]∆,[64]∆,[65]∆,[66]∆
	*Prunus cornuta* (Wall. ex Royle) Steud.	ISI-HS-57	Kala kath	Tree	Fruit	Eaten raw, Cooked	Digestive disorder, body tonic	9	0.04	2	0.22	78	2.1	[48]∆,[23]∆,[49]∆,[50]∆,[51]∆,[52]∆,[53]∆,[43]∆,[54]∆,[55]∆,[56]∆,[57]∆,[58]∆,[59]^®^,[60]∆,[61]∆,[62]∆,[63]∆,[64]∆,[65]∆,[66]∆
	*Prunus jacquemontii* Hook.f.	ISI-HS-51		Shrub	Fruit	Juice	Yellow fever, **eye infection**	22	0.09	2	0.09	68	4.6	[48]∆,[23]∆,[49]∆,[50]∆,[51]∆,[52]∆,[53]∆,[43]∆,[54]∆,[55]∆,[56]∆,[57]∆,[58]∆,[59]∆,[60]∆,[61]∆,[62]∆,[63]∆,[64]∆,[65]∆,[66]∆
	*Prunus persica* (L.) Batsch	ISI-HS-28	Aru	Tree	Fruit	Eaten raw	Coughs, asthma, **menstrual disorders**, constipation	34	0.15	4	0.12	79	14.2	[48]∆,[23]∆,[49]∆,[50]∆,[51]∆,[52]∆,[53]∆,[43]∆,[54]∆,[55]∆,[56]∆,[57]∆,[58]∆,[59]^®^,[60]^®^,[61]∆,[62]∆,[63]∆,[64]∆,[65]∆,[66]∆,
	*Prunus eburnean* Aitch.	ISI-HS-21	Burmi	Tree	Fruit	Eaten raw	Cancer, old wounds	19	0.08	2	0.11	63	4	[48]∆,[23]∆,[49]∆,[50]∆,[51]∆,[52]∆,[53]∆,[43]∆,[54]∆,[55]∆,[56]∆,[57]∆,[58]∆,[59]∆,[60]∆,[61]∆,[62]∆,[63]∆,[64]∆,[65]∆,[66]∆
	*Pyrus communis* L.	ISI-HS-13	Batang	Tree	Fruit	Juice	Diabetes, constipation	23	0.1	2	0.09	70	4.8	[48]∆,[23]∆,[49]∆,[50]∆,[51]∆,[52]∆,[53]∆,[43]∆,[54]∆,[55]∆,[56]∆,[57]∆,[58]∆,[59]∆,[60]∆,[61]∆,[62]^®^,[63]∆,[64]∆,[65]∆,[66]∆
	*Pyrus lanata* D.Don	ISI-HS-36	Doda	Tree	Fruit	Poultice	Urethritis, **cough** external wounds	14	0.06	3	0.21	50	4.3	[48]^®^,[23]∆,[49]∆,[50]∆,[51]∆,[52]∆,[53]∆,[43]∆,[54]∆,[55]∆,[56]∆,[57]∆,[58]∆,[59]∆,[60]∆,[61]∆,[62]∆,[63]∆,[64]∆,[65]∆,[66]∆
	*Pyrus pashia* Buch.-Ham. ex D.Don	ISI-HS-50	Batangi	Tree	Fruit	Paste, Poultice	Diarrhea, **dysentery**, wounds,	12	0.05	3	0.25	67	3.6	[48]∆,[23]^®^,[49]∆,[50]∆,[51]∆,[52]^®^,[53]^®^,[43]∆,[54]∆,[55]∆,[56]∆,[57]∆,[58]∆,[59]∆,[60]∆,[61]∆,[62]∆,[63]∆,[64]∆,[65]∆,[66]∆
	*Pyrus sinensis* Hemsl.	ISI-HS-56	Batangi		Fruit	Eaten raw	Skin allergy, asthma, hay fever,	16	0.07	3	0.19	81	4.8	[48]∆,[23]∆,[49]∆,[50]∆,[51]∆,[52]∆,[53]∆,[43]∆,[54]∆,[55]∆,[56]∆,[57]∆,[58]∆,[59]∆,[60]∆,[61]∆,[62]∆,[63]∆,[64]∆,[65]∆,[66]∆
	*Rosa macrophylla* Lindl.	ISI-HS-66		Shrub	Fruit, Root	Decoction	Eye infection, skin disease	18	0.08	2	0.11	61	4.2	[48]∆,[23]∆,[49]∆,[50]∆,[51]∆,[52]∆,[53]∆,[43]∆,[54]∆,[55]∆,[56]∆,[57]∆,[58]∆,[59]∆,[60]∆,[61]∆,[62]∆,[63]∆,[64]^®^,[65]∆,[66]∆
	*Rosa moschata* Herrm.	ISI-HS-23		Shrub	Fruit, Flower	Decoction	Skin disease, diarrhea, eye diseases, stomach disorder	36	0.15	4	0.11	81	15.1	[48]∆,[23]^®^,[49]∆,[50]∆,[51]∆,[52]∆,[53]∆,[43]^®^,[54]∆,[55]∆,[56]∆,[57]∆,[58]∆,[59]∆,[60]∆,[61]∆,[62]∆,[63]∆,[64]∆,[65]∆,[66]∆
	*Rosa webbiana* Wall. ex Royle	ISI-HS-52	Jangligulab	Shrub	Fruit, Seed	Juice	Digestive disorder, **jaundice**, blood purification	31	0.13	3	0.1	77	9.4	[48]∆,[23]∆,[49]∆,[50]∆,[51]∆,[52]∆,[53]∆,[43]∆,[54]∆,[55]∆,[56]∆,[57]∆,[58]^®^,[59]^®^,[60]∆,[61]∆,[62]∆,[63]∆,[64]∆,[65]^®^,[66]∆
	*Rubus ellipticus* Sm.	ISI-HS-67	PeelaGaracha	Shrub	Fruit	Juice	Jaundice, **fever**	11	0.05	2	0.18	82	2.3	[48]∆,[23]^®^,[49]∆,[50]∆,[51]∆,[52]^®^,[53]∆,[43]∆,[54]∆,[55]∆,[56]∆,[57]∆,[58]∆,[59]∆,[60]∆,[61]∆,[62]∆,[63]∆,[64]∆,[65]∆,[66]∆
	*Rubus fruticosus* L.	ISI-HS-47	Kala Garacha	Shrub	Leaves, Fruit	Infusion or decoction	Diarrhea, heart diseases, anemia	28	0.12	3	0.11	75	7.8	[48]∆,[23]∆,[49]∆,[50]∆,[51]∆,[52]∆,[53]∆,[43]∆,[54]∆,[55]∆,[56]∆,[57]∆,[58]∆,[59]∆,[60]^®^,[61]^®^,[62]∆,[63]∆,[64]∆,[65]∆,[66]∆
	*Rubus hoffmeisterianus* Kunth&C.D.Bouché	ISI-HS-14	SurkhGaracha	Shrub	Leaves, Fruit	Paste	Skin diseases, **stomach disorder**, jaundice,	30	0.13	3	0.1	77	9.1	[48]∆,[23]∆,[49]∆,[50]∆,[51]∆,[52]∆,[53]∆,[43]∆,[54]∆,[55]∆,[56]∆,[57]∆,[58]∆,[59]∆,[60]∆,[61]∆,[62]∆,[63]∆,[64]^®^,[65]∆,[66]∆
	*Rubus sanctus* Schreb.	ISI-HS-4	Garacha	Shrub	Fruit	Decoction	Eye infection, **skin disease**, diarrhea	15	0.06	3	0.2	80	4.6	[48]∆,[23]∆,[49]∆,[50]∆,[51]∆,[52]∆,[53]∆,[43]∆,[54]∆,[55]∆,[56]∆,[57]∆,[58]∆,[59]∆,[60]∆,[61]∆,[62]∆,[63]^®^,[64]∆,[65]∆,[66]∆
	*Spiraea hazarica* R.Parker	ISI-HS-18		Herb	Fruit, Leaves	Decoction,	Liver disorders, cold, sthroat disorder	29	0.12	3	0.1	76	8.7	[48]∆,[23]∆,[49]∆,[50]∆,[51]∆,[52]∆,[53]∆,[43]^®^,[54]∆,[55]∆,[56]∆,[57]∆,[58]∆,[59]∆,[60]∆,[61]∆,[62]∆,[63]∆,[64]∆,[65]∆,[66]∆
	*Spiraea vaccinifolia* D.Don	ISI-HS-69		Shrub	Fruit, Leaves	Infusion, Juice	Joint pain, constipation	13	0.06	2	0.15	62	2.7	[48]∆,[23]∆,[49]∆,[50]∆,[51]∆,[52]∆,[53]∆,[43]^®^,[54]∆,[55]∆,[56]∆,[57]∆,[58]∆,[59]∆,[60]∆,[61]∆,[62]∆,[63]∆,[64]∆,[65]∆,[66]∆
Rutaceae	*Citrus maxima* (Burm.) Merr.	ISI-HS-73	Chakotra	Tree	Leaves, Fruit	Decoction, Powder	Heart tonic, **body swellings**	19	0.08	2	0.11	63	6.6	[48]∆,[23]∆,[49]^®^,[50]∆,[51]∆,[52]∆,[53]∆,[43]∆,[54]∆,[55]∆,[56]∆,[57]∆,[58]∆,[59]∆,[60]∆,[61]∆,[62]∆,[63]∆,[64]∆,[65]∆,[66]∆
	*Zanthoxylum armatum* DC.	ISI-HS-37	Timar	Climber	Fruit	Powder	Stomachic, improve digestion	17	0.07	2	0.12	59	4	[48]^®^,[23]^®^,[49]∆,[50]∆,[51]∆,[52]∆,[53]^®^,[43]^®^,[54]∆,[55]∆,[56]∆,[57]∆,[58]∆,[59]∆,[60]∆,[61]∆,[62]∆,[63]∆,[64]∆,[65]∆,[66]∆
Solanaceae	*Solanum surattense* Burm.f.	ISI-HS-53	Mara ghinrhye	Shrub	Fruit	Decoction, Powder	Jaundice, **diabetes**, blood purification, asthma	34	0.15	4	0.12	79	14.2	[48]∆,[23]∆,[49]^®^,[50]∆,[51]∆,[52]∆,[53]∆,[43]∆,[54]∆,[55]∆,[56]∆,[57]∆,[58]∆,[59]∆,[60]∆,[61]∆,[62]∆,[63]∆,[64]∆,[65]∆,[66]^®^
Thymelaeacee	*Daphne mucronata* Royle	ISI-HS-72	Luni	Shrub	Leaves	Powder, Paste	Muscle pains, women infertility, infectious wounds, menstruation disorders, gynaecological, infections, constipation, skin diseases	42	0.18	7	0.17	100	30.4	[48]∆,[23]∆,[49]∆,[50]∆,[51]∆,[52]∆,[53]∆,[43]^®^,[54]∆,[55]∆,[56]∆,[57]∆,[58]^®^,[59]∆,[60]∆,[61]∆,[62]∆,[63]∆,[64]∆,[65]^®^,[66]∆
Urticaceae	*Debregeasia saeneb* (Forssk.) Hepper & J.R.I.Wood	ISI-HS-71	Chainjal	Shrub	Fruit	Eaten raw	Diarrhea, **constipation**, body tonic	25	0.11	3	0.12	72	8.1	[48]∆,[23]∆,[49]∆,[50]∆,[51]∆,[52]∆,[53]∆,[43]∆,[54]∆,[55]∆,[56]∆,[57]∆,[58]∆,[59]∆,[60]∆,[61]∆,[62]∆,[63]∆,[64]∆,[65]∆,[66]∆
Vitaceae	*Vitis vinifera* L.	ISI-HS-68	Angoor	Climber	Fruit	Powder, Paste	Dyspepsia, **constipation**	20	0.09	2	0.1	65	4.2	[48]∆,[23]∆,[49]^®^,[50]∆,[51]^®^,[52]∆,[53]∆,[43]∆,[54]∆,[55]∆,[56]∆,[57]∆,[58]∆,[59]∆,[60]^®^,[61]∆,[62]∆,[63]^®^,[64]∆,[65]∆,[66]∆
	*Vitis jacquemontii* R.Parker	ISI-HS-54	Angoor	Shrub	Fruit	Eaten raw	Body tonic, constipation	18	0.08	2	0.11	61	4.2	[48]∆,[23]^®^,[49]∆,[50]∆,[51]∆,[52]∆,[53]∆,[43]∆,[54]∆,[55]∆,[56]∆,[57]∆,[58]∆,[59]∆,[60]∆,[61]∆,[62]∆,[63]∆,[64]∆,[65]∆,[66]∆
	*Vitis flexuosa* Thunb	ISI-HS-49	Angoor	Climber	Fruit	Powder	Typhoid, colds	14	0.06	3	0.21	71	3.3	[48]∆,[23]∆,[49]∆,[50]^®^,[51]∆,[52]∆,[53]∆,[43]∆,[54]∆,[55]∆,[56]∆,[57]∆,[58]∆,[59]∆,[60]∆,[61]∆,[62]∆,[63]∆,[64]∆,[65]∆,[66]∆

(♦) = plant with similar use(s); ^®^ = plant with dissimilar use(s); (∆) = plant not reported in previous study.

**Table 3:** ICF values and diseases categories in study are.

Category of Diseases	Number of Use Reports	% of Use Reports	Number of Taxa Used	% of Taxa	ICF
GIT diseases	59	53.2	44	59.5	0.26
Respiratory diseases	18	16.2	14	18.9	0.24
Muscles and skeletal disorders	16	14.4	13	17.6	0.20
Urinary disorders	10	9.0	9	12.2	0.11
Sexual diseases	9	8.1	6	8.1	0.38
Glandular disorders	23	20.7	19	25.7	0.18
Ear, nose, eyes diseases	9	8.1	9	12.2	0.00
Nail, skin, and hair disorders	12	10.8	11	14.9	0.09
Nervous disorders	8	7.2	7	9.5	0.14
Cardiovascular disorders	25	22.5	23	31.1	0.08
Body energizers	12	10.8	11	14.9	0.09

## Data Availability

The raw data contain the names of all participants and cannot be shared in this form.
